# Supplementary material for: Morphology of Meibomian Glands in a 65-Year-Old Norwegian Population without Dry Eye Disease
Source: J Clin Med. 2022 Jan 20;11(3):527. doi: 10.3390/jcm11030527 (PMC8836511; doi:10.3390/jcm11030527)
Supplement: Supplementary file 1 [file jcm-11-00527-s001.zip › jcm-1547565-supplementary.pdf]

Table S1. Correlation between meibomian gland morphology and clinical dry eye tests.

		OSDI	Osmolarity	TFBUT	OSS	Schirmer I	ME	MQ	LMA
Precent dropout_UL	*r*	-0.051	0.094	-0.137	.246*	-0.126	0.002	-0.025	0.147
	p	0.603	0.537	0.172	0.012	0.203	0.988	0.802	0.135
Percent dropout_LL	*r*	-0.153	-.353*	0.011	-0.047	-.206*	-0.026	0.026	-0.088
	p	0.102	0.011	0.905	0.621	0.029	0.783	0.786	0.348
Percent dropout_middle_UL	*r*	0.007	0.202	0.054	0.132	-0.071	-0.03	-0.011	0.034
	p	0.942	0.177	0.593	0.18	0.476	0.761	0.913	0.729
Percent dropout_middle_LL	*r*	-0.091	-0.197	0.139	0.013	-0.09	-0.133	0.072	-0.084
	p	0.334	0.165	0.145	0.889	0.34	0.158	0.448	0.37
Number of glands_UL	*r*	-0.006	-.328*	0.165	-.255**	0.051	0.033	-0.025	-0.141
	p	0.952	0.026	0.099	0.009	0.606	0.737	0.798	0.153
Number of glands_LL	*r*	-0.183	-0.177	-.254**	-0.027	-0.004	.255**	-.193*	0.01
	p	0.05	0.214	0.007	0.773	0.968	0.006	0.04	0.916
Distorted glands_UL	*r*	0.059	0.054	0.006	-0.025	0.074	0.15	-0.177	-0.106
	p	0.55	0.723	0.955	0.803	0.459	0.128	0.072	0.284
Distorted glands_LL	*r*	0.102	-0.097	-0.1	-0.076	0.006	-0.003	0.103	-0.055
	p	0.28	0.498	0.297	0.417	0.95	0.975	0.278	0.562
Tortuous glands_UL	*r*	-0.028	-0.033	0.152	-.312**	0.022	-.206*	0.074	-0.049
	p	0.776	0.828	0.129	0.001	0.828	0.036	0.453	0.62
Tortuous glands_LL	*r*	.213*	0.17	0.092	-.199*	0.099	-0.023	0.027	-0.08
	p	0.022	0.233	0.335	0.033	0.297	0.804	0.773	0.397
Hooked glands_UL	*r*	-0.035	-0.054	-0.032	-0.092	-0.098	-0.127	0.087	0.184
	p	0.72	0.72	0.748	0.353	0.324	0.2	0.38	0.061
Hooked glands_LL	*r*	-0.038	0.019	-0.019	0.047	0.043	-0.048	0.125	0.003
	p	0.688	0.895	0.84	0.62	0.653	0.612	0.185	0.973
Dropout glands_UL	*r*	-.196*	-0.031	-0.114	0.086	-0.185	0.109	-0.04	-0.087

	p	0.046	0.838	0.255	0.381	0.062	0.27	0.686	0.377
Dropout glands_LL	*r*	-0.002	0.007	0.123	0.162	-0.032	-0.142	0.044	0.141
	p	0.983	0.962	0.199	0.084	0.735	0.131	0.646	0.133
Shortened glands_UL	*r*	0.029	0.019	-0.04	0.093	-0.028	0.107	-0.138	0.008
	p	0.767	0.898	0.691	0.345	0.782	0.279	0.161	0.934
Shortened glands_LL	*r*	-0.063	-.300*	-0.001	0.078	-.198*	0.032	-0.011	-0.065
	p	0.507	0.032	0.993	0.41	0.036	0.736	0.91	0.491
Overalapping glands_UL	*r*	-0.059	-0.195	0.145	-.264**	0.104	0.031	-0.074	-0.122
	p	0.551	0.195	0.147	0.006	0.296	0.758	0.455	0.216
Overalapping glands_LL	*r*	-0.171	-0.03	-0.1	-0.12	0.048	-0.007	0.009	-0.016
	p	0.068	0.836	0.295	0.201	0.612	0.943	0.928	0.863
Ghost glands_UL	*r*	-0.054	0.087	0.064	-0.03	-0.067	0.167	-0.128	-0.085
	p	0.586	0.566	0.523	0.762	0.502	0.091	0.197	0.388
Ghost glands_LL	*r*	-0.03	-0.086	0.142	-0.1	-0.107	0.052	-0.12	0.103
	p	0.751	0.549	0.138	0.289	0.258	0.583	0.205	0.273
Tadpoling_UL	*r*	.222*	-0.127	0.047	0.054	-0.016	0.148	-0.061	-0.013
	p	0.022	0.401	0.639	0.586	0.873	0.131	0.539	0.892
Tadpoling_LL	*r*	0.047	-0.266	0.011	-0.147	-0.154	0.064	-0.139	0.103
	p	0.618	0.059	0.909	0.117	0.103	0.496	0.14	0.273
Abnormal gap_UL	*r*	-0.044	0.028	0.125	-.223*	-0.089	-0.024	0.041	-0.115
	p	0.658	0.853	0.215	0.022	0.372	0.811	0.682	0.243
Abnormal gap_LL	*r*	0.169	0.096	0.048	-0.072	-0.071	-0.011	0.169	0.022
	p	0.07	0.504	0.614	0.444	0.454	0.91	0.072	0.815
Fluffy areas_UL	*r*	0.051	-0.282	0.092	-0.143	-0.02	-0.094	0.049	-0.061
	p	0.602	0.058	0.36	0.146	0.841	0.341	0.621	0.54
Fluffy areas_LL	*r*	0.096	.	0.155	-0.073	-0.064	-0.121	0.086	0.042
	p	0.308	.	0.105	0.436	0.502	0.2	0.364	0.655
No extention to lid margin_UL	*r*	-0.114	-0.152	0.044	-0.058	0.096	0.03	0.016	-0.105

	p	0.247	0.314	0.663	0.554	0.337	0.764	0.871	0.287
No extention to lid margin_LL	*r*	-0.053	-0.047	-0.174	-0.085	0.037	0.11	0.035	-0.019
	p	0.571	0.741	0.067	0.368	0.697	0.242	0.709	0.841

UL= upper lids; LL= lower lids; TFBUT= tear film break-up time; OSS= ocular surface staining; ME= meibum expressibility; MQ= meibum quality; LMA= limbal margin abnormality score. *P-values from Spearman rank correlation analysis.
